# Driving Factors and Spatiotemporal Characteristics of CO_2_ Emissions from Marine Fisheries in China: A Commonly Neglected Carbon-Intensive Sector

**DOI:** 10.3390/ijerph20010883

**Published:** 2023-01-03

**Authors:** Xiao Zhang, Shengchao Ye, Manhong Shen

**Affiliations:** 1 School of Business, Ningbo University, Ningbo 315211, China; 2 School of Economics and Management, Zhejiang A&F University, Hangzhou 311300, China

**Keywords:** marine fisheries, CO_2_ emissions, Kernel density estimation, spatial Durbin model, spatial spillover effect

## Abstract

The CO_2_ emissions from marine fisheries have a significant impact on marine ecology, despite generally being overlooked in studies on global climate change. Few studies have estimated the carbon emissions from marine fisheries while taking into account all pertinent sectors. This study evaluated marine fisheries’ CO_2_ emissions based on three sectors: marine fishing, mariculture, and the marine aquatic product processing industry. Kernel density estimation and the spatial Durbin model were used to investigate the spatial and temporal characteristics and the key socioeconomic drivers of the CO_2_ emissions from marine fisheries in 11 coastal provinces of China from 2005 to 2020. The results are as follows: (1) marine fishing is the sector that produces the most CO_2_ emissions; trawling operations generate more CO_2_ than all other modes of operation combined; (2) China’s marine fisheries’ CO_2_ emissions show a rising, then declining, trend, with significant differences in coastal provinces; (3) the development of the marine fishery economy and trade have a positive driving effect on CO_2_ emissions, the expansion of the tertiary industry does not decrease CO_2_, the technical advancement and income growth of fishermen are negatively related to carbon emissions, and the effect of environmental regulation has failed to pass the significance test; (4) the carbon emissions of marine fisheries have significant spatial spillover effects.

## 1. Introduction

Climate change poses several unpredictable challenges to marine ecosystems; according to the World Meteorological Organization’s State of the Global Climate 2021 report, the Earth’s greenhouse gases have reached an all-time high, and four key climate change indicators—greenhouse gas concentration, sea level rise, ocean heat, and ocean acidification—have all hit new records. Climate change has become a major challenge to human development and has been dubbed “the largest market failure the world has ever seen”. Maintaining the increase in global temperature within 1.5 °C and lowering global greenhouse gas emissions have become crucial concerns for all nations, putting high-carbon countries such as China under enormous pressure [1,2]. In broad industry decarbonization studies, marine fisheries’ carbon emissions are often excluded from global GHG assessments. The fact is, however, that the ocean can no longer receive any more CO_2_ [3]. Global marine fisheries’ carbon emissions have irreversible direct effects on the marine ecosystem [4], including ocean acidification [5,6], sea level rise [7], and biodiversity loss [8,9,10,11], while also jeopardizing food security, human health, and other socioeconomic concerns [12,13,14,15]. This also poses a greater challenge to the allocation of marine resources among countries and regions, as sudden increases or decreases in catches may trigger conflicts between areas [16,17]. In summary, CO_2_ can damage natural systems by altering ocean-derived resources, which in turn affects human well-being and economic order [18].

Large amounts of CO_2_ are produced not only by the marine fishing sector, but also the mariculture and marine aquatic product processing industries. China’s coastal areas have long been engaged in high-energy marine activities. China is responsible for 15% of all marine fishing activities worldwide and has the largest mariculture sector in the world. Additionally, China is the largest importer and exporter of fishery goods. China imports fishery products not only for local consumption, but also as raw materials to be processed and re-exported. Consequently, the mariculture and marine product processing industries contribute substantial carbon emissions as well.

As the international framework for climate policy emphasizes the concept of “shared but differentiated duties,” and China is a big producer and emitter of carbon from marine fisheries, it must aggressively begin to lower emissions in this sector [19]. China has always participated in and contributed to the global response to climate change, and several sectors are exerting substantial efforts to promote carbon reduction goals [20,21]. However, the marine fishery industry has not yet been incorporated into China’s main emission reduction inventory and carbon trading programs. In addition, there are no universal accounting standards for carbon emissions from marine fisheries, nor is there any systematic research on CO_2_ emission sources, drivers, or trends. Consequently, the trajectory of carbon emissions from China’s marine fishery fuels must be rigorously monitored, and an in-depth study on the influencing elements must be conducted to investigate a development path of decarbonization for marine fisheries in China.

The contributions of this paper are as follows. We first propose a CO_2_ accounting paradigm that can more effectively address substantial measurement errors and the insufficient sectoral coverage of marine fisheries in prior research. Following that, kernel density analysis is used to clarify the dynamic evolution of CO_2_. Subsequently, on the basis of coastal province panel data, a spatial Durbin model (SDM) is used to analyze the key factors affecting changes in carbon emissions from marine fisheries in China and to explore whether there is a spatial spillover effect. The findings provide crucial inspiration and a scientific foundation for designing policies to effectively restrict China’s marine fisheries’ CO_2_ emissions increase.

## 2. Literature Review

The oceans, as climate integrators, have absorbed 28% of the global CO_2_ emissions since 1750, offsetting most of the atmospheric warming [3]. Based on historical catches and fuel usage, Mariani, G. et al. [22] estimate that marine fisheries have released at least 730 million tons of CO_2_ into the atmosphere since 1950. Ferrer, E. [23] points out that the global emissions of marine fishing have increased by 28% (an average of 21% per ton of catch) from 1990 to 2011, but production has barely increased in parallel. Parker, R. [24] calculated the total global CO_2_ emissions from the industrial fishing sector in his Ph.D. thesis using a database of fuel and energy usage including 1126 records of global catches. According to the study, fishing activities consumed 40 billion liters of fuel in 2011 and released 168 million tons of CO_2_ equivalent into the environment [25]. Greer, K. et al. [26] studied the global CO_2_ emissions from marine fisheries using the Global Marine Fishing Effort Database, which provides the CO_2_ emissions and carbon intensity (CO_2_ emission per unit catch) of global marine fisheries from 1950 to 2016 and found that CO_2_ industrial fisheries released 39 million tons of CO_2_ in 1950, with this figure increasing to 159 million tons in 2016, much lower values than those found by Parker. The reason for this discrepancy is mainly that both studies included a significant number of unreported data and utilized different approaches for handling missing values. However, both Greer’s and Parker’s efforts reveal that China’s marine fisheries produce the highest CO_2_ emissions.

China’s marine fisheries have the highest carbon intensity and overall volume, accounting for almost one-third of global CO_2_ emissions from marine fisheries and exceeding those of all European and American fisheries combined [27]. Furthermore, China’s marine fisheries are extremely vulnerable to climate change. Robert noted in his analysis of marine fisheries in 147 countries, via measuring their climate change vulnerability index, that China ranked 8th and possessed high vulnerability. Nearly all of the top 10 most vulnerable countries had a low carbon emission intensity, but only China showed both high carbon intensity and high vulnerability [28]. Studies conducted by Chinese scientists mostly focus on estimating energy consumption from fishing vessel operations. In 2007 and 2008, the CO_2_ emissions from fossil fuel combustion by marine fishing vessels in China totaled 20.1311 and 24.706 million tons, respectively [29,30]. In a more recent study, Wu, J. and Li, B. [31] calculated that CO_2_ emissions were primarily caused by indirect emissions, which is perhaps a debatable result. Regardless of the research cited above, China has long been the largest source of carbon emissions in the world’s marine fisheries; this also indicates that China has a great deal of room to cut emissions [32].

From the perspective of influencing factors, CO_2_ emissions from marine fisheries are directly tied to fuel consumption, and fuel consumption by fishing vessels has been demonstrated to be an appropriate indicator for measuring CO_2_ emissions from fishing activities [33,34,35]. Fuel is needed to propel the vessel, handle the catch on board and freeze it, and provide electricity for the fishermen on board [36]. Other upstream fishing processes, such as vessel construction and maintenance, gear manufacturing, and bait supply, as well as downstream post-landing activities such as processing, packing, and transportation, all consume energy and produce emissions [37]. Concomitantly, fishing vessel fuel continues to be the most significant source of carbon emissions in the seafood supply chain, accounting for approximately 96% of the overall process carbon footprint [38]. Meanwhile, fuel accounts for the second highest expense after labor.

Carbon emissions from marine fisheries are also a result of a variety of economic decisions. In the context of fuel subsidies and growing fuel prices, academics are increasingly focusing on the control of fishery activities [39,40,41]. Offsetting fuel costs is the primary goal of many governments around the world when implementing fishery subsidies, and government intervention in this area is also very strong in developed countries [42]. By evaluating the association between energy use and catch rates, quotas, and oil prices, Schau, E. et al. [43] discovered a long-term negative correlation between fish fuel consumption and annual catch rates as well as oil prices. Furthermore, the different marine fishery operation modes of fishing vessels produce varying amounts of carbon emissions, with trawling being the most carbon-intensive [44,45].

In summary, marine fisheries contribute various sources of emissions, and the CO_2_ emissions from this system comprise not only the use of fossil fuels by marine fishing boats, but also those from other industrial operations. However, research on marine fisheries is currently in the early stages, and the pertinent study findings are still ambiguous. Studies on carbon emissions from marine fisheries are mostly concentrated on the subject of capture fisheries, with the mariculture and marine product processing sectors receiving less attention. In terms of study methodologies, the majority of earlier analyses of the variables impacting CO_2_ in marine fisheries have focused on energy use, economic development, and industrial size, which may present independent variable multiple linear problems. In comparison with previous studies, this paper develops a more detailed method for calculating the CO_2_ produced by marine fisheries by considering all sectors and uses a spatial econometric model to systematically examine the driving effects of geographic, social, economic, and policy factors on CO_2_.

## 3. Methodology and Data

### 3.1. Calculation Framework and Data

Accounting for carbon emissions is crucial for carbon reduction activities, and only by quantifying the carbon emission levels of all stakeholders can collaborative carbon reduction measures be implemented in coastal provinces and diverse marine fisheries sectors. The framework for measuring CO_2_ emissions from marine fisheries is as follows:

1. CO_2_ emissions from the marine fishing sector mainly come from the consumption of diesel fuel by capture fishing vessels;
2. CO_2_ emissions from the mariculture sector come from two sources: first, the consumption of diesel fuel by mariculture fishing vessels, and second, the consumption of electricity by oxygen supply and electric pumps in mariculture ponds and industrial farming;
3. CO_2_ emissions from the marine aquatic product processing sector mainly come from the electricity consumed by cold storage and processing. The formula is as follows:

(1) $$TC_{fishery}=C_{mf}+C_{mc}+C_{mp}$$

(2) $$C_{mf}=\sum^{\;}(E_{itp}^{diesel\_mf\;}\cdot f\cdot r\cdot c\cdot o\cdot 44/12)$$

(3) $$E_{ipt}^{diesel\_mf\;}=\sum_{j=1}^{6}(E_{it}^{power\_mf\;}\cdot \delta_{p}^{mf})$$

(4) $$C_{mc}=\sum^{\;}(E_{it}^{diesel\_mc\;}\cdot \delta^{mc}\cdot f\cdot r\cdot c\cdot o\cdot 44/12)+\sum^{\;}(E_{it}^{electric\_mc\;}\cdot \theta_{i})$$

(5) $$C_{mp}=\sum^{\;}(E_{it}^{electric\_cold\_store}\cdot \theta_{i})+\sum^{\;}(E_{it}^{electric\_process}\cdot \theta_{i})$$

Table 1 shows the meaning of the symbols in Equations (1)–(5). The methods for the calculation of CO_2_ emissions from the marine aquatic product processing industry were obtained from reference [46].The coefficients used to calculate the carbon emissions of fishing vessels are from the “2006 IPCC Guidelines for National Greenhouse Gas Inventories”; $\delta_{j}^{mf}$ was derived from the “Reference Standard for Measurement of Oil Consumption for China’s Motor Fishing Vessel Oil Price Subsidy”; $\theta_{i}$ was obtained from the “CO_2_ Emission Accounting Methodology and Data Verification Table” issued by the Climate Department of the Chinese Ministry of Ecology and Environment. Other original data for this article were obtained from the China Fishery Statistical Yearbook, Statistical Yearbook of Import and Export Trade of Aquatic Products in China, China Marine Economy Yearbook, and the China Energy Yearbook. The interpolation method and GDP growth rate inversion method were mostly used to supplement missing data.

**Figure 1:**
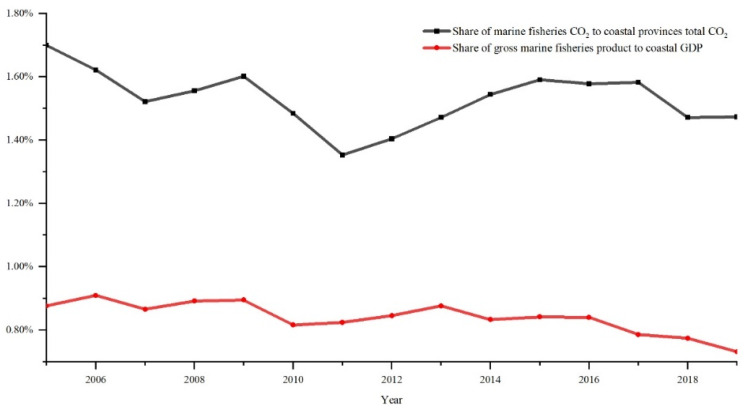
Comparison of the share of gross marine fisheries product to coastal GDP and the share of marine fisheries CO_2_ to total CO_2_ in coastal provinces. Note: Total CO_2_ data from https://www.ceads.net.cn, accessed on 28 December 2022.

Figure 1 shows the comparison between the share of gross marine fisheries product to coastal GDP and the share of marine fisheries CO_2_ to total CO_2_ in coastal provinces. The average value for the contribution of the marine fishery economy to the coastal GDP is 0.84% and holds a decreasing trend. However, the average ratio of CO_2_ emissions from marine fisheries to total emissions is 1.53%, which is significantly higher than the economic share. That suggests that China’s marine fisheries sector is in extensive growth, and the negative externalities are highly apparent. Marine fisheries do not produce a significant amount of CO_2_ when compared to industrial activity. However, given that diesel engines will continue to be the primary source of propulsion for fishing boats for a prolonged period, as well as the high cost and resistance of improved fuel, these make the decoupling of carbon emissions a challenge. The CO_2_ issue may possibly pose a significant obstacle to the economic growth of marine fisheries in China; thus, a thorough investigation of CO_2_ variations and drivers of marine fisheries CO_2_ is required.

### 3.2. Variable Description

Carbon emissions are a byproduct of general economic activity; hence, carbon emissions from marine fisheries’ fuel consumption are a result of various economic decisions. Measuring, monitoring, managing, and mitigating carbon emissions from fisheries necessitate a deeper understanding of the drivers of carbon emissions and the socioeconomic uncertainties encountered in the carbon reduction process in order to manage carbon emissions from an economy at the macro level. The independent variables selected for this paper are shown in Table 2.

It is worth emphasizing that the explained variable is denoted by *lnPCO_2_*, since the idea of per capita carbon emissions is more representative of socioeconomic features and equity [47]. The “environmental regulation” (*lnPOL*) variable was selected from the system of controlling the total number and power of marine fishing vessels in China. China has been implementing this approach since 1987, in response to the poor ecological effects of offshore fishing. However, 35 years have since passed, and the number and power of fishing vessels remain uncontrollably high. This article employs an econometric approach to investigate whether the policy has contributed to the development of the marine environment.

### 3.3. Model Settings

#### 3.3.1. Kernel Density Estimation

In order to formulate effective emission strategies for different coastal provinces, we need to sufficiently investigate the dynamic evolutionary characteristics of marine fisheries’ CO_2_ emissions. The Kernel density estimation method was used to estimate the dynamic evolution trend. As a nonparametric estimation method, it is able to describe the distribution location, distribution pattern, polarization trend, and ductility of random variables with continuous density curves, and then estimate the probability density of random variables under a limited sample [48,49]. In this paper, the more commonly used Gaussian kernel function is adopted for estimation [50,51], and the expression is as follows:

(6) $$\hat{f_{h}}\left(x\right)=\frac{1}{n}\sum_{i=1}^{n}K_{n}\left(x-x_{i}\right)=\frac{1}{nh}\sum_{i=1}^{n}K\left(\frac{x-x_{i}}{h}\right)$$

(7) $$k\left(x\right)=\frac{1}{\sqrt{2\pi }}\;\exp\;(-\frac{x^{2}}{2})$$

where f(x) is the density function, $x_{i}$ denotes the independent identically distributed observations, *k*(·) represents the Gaussian kernel function, and *h* is the bandwidth.

#### 3.3.2. Spatial Econometric Model

Considering that carbon dioxide will spread to surrounding areas with natural factors, such as atmospheric conditions, as well as socioeconomic factors, such as industrial structure, market trade, and technology spillovers, there will be a certain “convergence” effect on the carbon emission levels of neighboring provinces. The spatial effect of carbon emissions will be investigated in this article utilizing spatial econometric approaches. A spatial autocorrelation test should be performed prior to building the spatial econometric model. In this paper, Moran’s I is used to examine the spatial correlation of carbon emissions from marine fisheries among provinces with the following equation:

(8) $$Moran’s\;I=\frac{\sum_{i=1}^{n}\sum_{j=1}^{n}w_{ij}\left(x_{i}-\bar{x}\right)\left(x_{j}-\bar{x}\right)}{\sum_{i=1}^{n}{\left(x_{i}-\bar{x}\right)}^{2}}$$

where $x_{i}$ is the observed value of per capita carbon emissions from marine fisheries in each province, and $w_{ij}$ is the spatial weight. In this paper, the spatial weight matrix is adopted as the spatial geo-economic weight matrix, the formula for which is as follows:

(9) $$W_{ij}=\{\begin{array}{c} \frac{\left|\begin{array}{c} \bar{Y_{i}} \end{array}-\bar{Y_{j}}\right|}{d_{ij}^{2}}\;\mathrm{if}\;i\neq j\\ 0\;\mathrm{if}\;i=j \end{array}$$

$Y_{i}$, $Y_{j}$ denote the difference in GDP per capita of region *i* relative to region *j*, and the distance $d_{ij}$ between the two provinces is obtained from the latitude and longitude coordinates of the geographic centers of provinces *i* and *j.*

**Figure 2:**
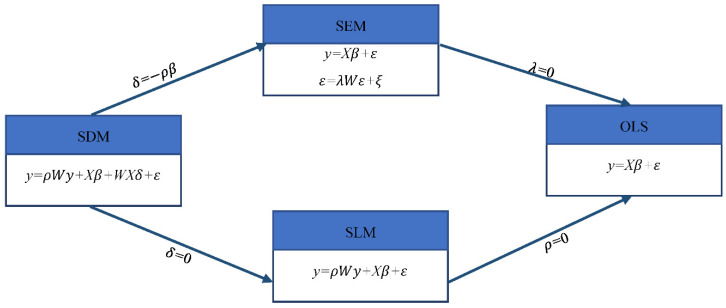
Transfer among spatial econometric models.

Anselin, L. [52] proposed two spatial autocorrelation models in 1988, the spatial lag model (SLM) and the spatial error model (SEM). The SLM model focuses on whether there is a diffusion of each variable in the same region, and the SEM model mainly studies the effect of the error of the dependent variable in the neighboring region on the regional observations. In 2009, Lesage, J. et al. [53] constructed a spatial Durbin model (SDM) based on Anselin’s study. The transformation among models is shown in Figure 2. In particular, the SDM contains both dependent and explanatory variable lagged terms, which could help reduce bias due to omitted variables in the empirical analysis.

The spatial error model (SEM), spatial lag model (SLM), and spatial Durbin model (SDM) are established in this paper, Equations (10)–(12). The optimal models were determined based on the LR, LM, and WALD tests.

(10) $$lnPCO2_{it}=\beta_{1}lnGMFP_{it}+\beta_{2}lnINDUS_{it}+\beta_{3}lnTECH_{it}+\beta_{4}lnENERGY_{it}+\beta_{5}lnIMEX_{it}\;+\beta_{6}lnPOL_{it}+\mu_{i}+\gamma_{t}+\varepsilon_{it}\;\varepsilon_{it}=\lambda W_{i}\varepsilon_{t}+\xi_{it}$$

(11) $$lnPCO2_{it}=\rho W_{i}y_{t}+\;\beta_{1}lnGMFP_{it}+\beta_{2}lnINDUS_{it}+\beta_{3}lnTECH_{it}+\beta_{4}lnENERGY_{it}+\beta_{5}lnIMEX_{it}+\beta_{6}lnPOL_{it}\;+\mu_{i}+\gamma_{t}+\varepsilon_{it}$$

(12) $$lnPCO2_{it}=\rho W_{i}y_{t}+\beta_{1}lnGMFP_{it}+\beta_{2}lnINDUS_{it}+\beta_{3}lnTECH_{it}+\beta_{4}lnENERGY_{it}+\;\beta_{5}lnIMEX_{it}+\beta_{6}lnPOL_{it}+W_{i}X_{t}\delta +\mu_{i}+\gamma_{t}+\varepsilon_{it}$$

In this formula, $lnPCO2_{it}$ is the dependent variable, *c* is the constant term, $\beta_{1,2\cdots 6}$ denotes the parameter to be estimated for the independent variable, *ρ* is the spatial autoregressive coefficient, and $\xi_{it}$ is the idiosyncratic component, which can also be considered as the interference. *δ* is the spatial lag coefficient of the explanatory variables, $\mu_{i}$, $\gamma_{t}$ denote spatial and time effects, and $\varepsilon_{it}$ is the residual term. $W_{i}X_{t}$ represents the spatial spillover effect; that is, the influence of the independent variable of the neighboring region on the dependent variable of the local region.

## 4. Results and Discussion

### 4.1. Spatial and Temporal Characteristics of CO_2_ Emissions from Marine Fisheries

#### 4.1.1. CO_2_ Levels and Time-Varying Characteristics

**Figure 3:**
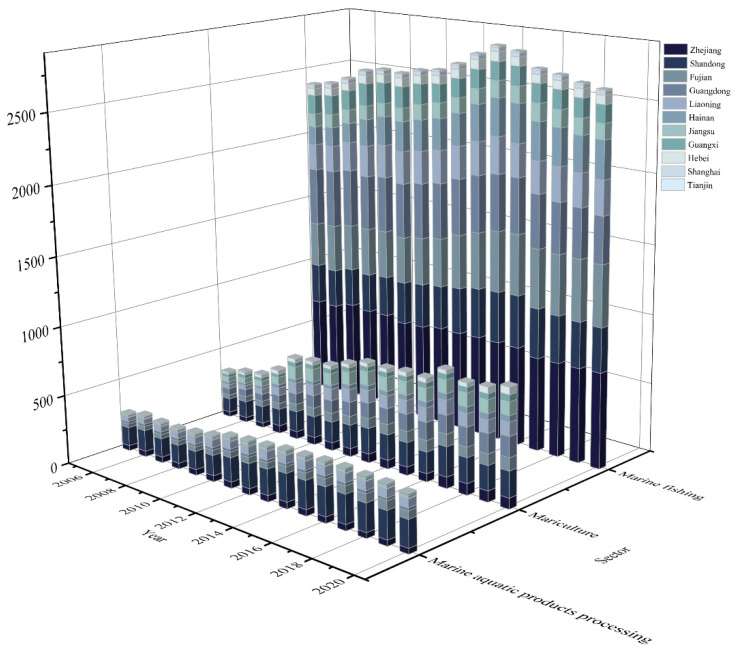
Marine fisheries’ CO_2_ emissions in three main sectors ($10^{4}$ t).

The total CO_2_ emissions in 2020 amounted to 38.81 million tons, which is 29% greater than the amount produced in 2005, and the carbon emissions from marine fisheries reached a peak in 2015, followed by a slight decline. The average amount of CO_2_ emissions in the study period was 36.47 million tons, which is higher than the value given by reference [24] but lower than [26]. The cumulative carbon emissions from marine fisheries in the three studied sectors from 2005 to 2020 are visualized in Figure 3. The marine fishing sector was found to be the main contributor of carbon emissions, accounting for 72% of total emissions, which is contrary to results in the literature [31]. Meanwhile, the share of CO_2_ emissions from the mariculture and marine aquatic product processing sectors is growing. In 2020, compared to 2005, mariculture increased by 141.23%, with the largest increase seen in the provinces of Guangdong, and Tianjin was the only province with a downward trend. The marine aquatic product processing sector is also continuously growing, with an increase of 45.34%. The largest CO_2_ emissions and increase were seen in Shandong Province, followed by Liaoning and other provinces with relatively small changes.

**Figure 4:**
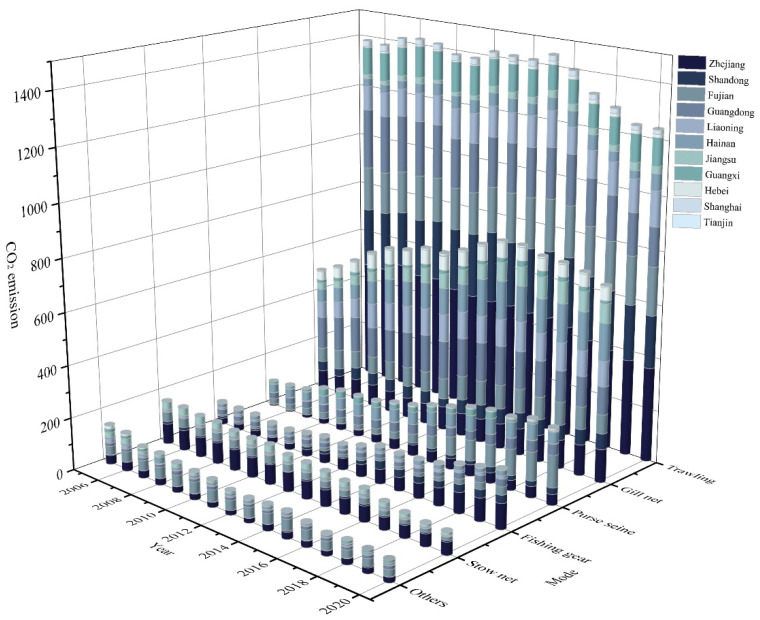
CO_2_ emissions of marine fishing industry in different operation modes ($10^{4}$ t).

Figure 4 shows the CO_2_ emissions of the marine fishing industry under different operation modes. Trawling produced the largest amount of CO_2_, accounting for 52.37% of emissions from the marine fishing industry, which is also consistent with the studies of [43]. Trawling and gillnet use have a significant negative ecological impact on marine fisheries. Trawling has a low goal catch rate, destroys biodiversity, and mobilizes centuries’ worth of carbon emissions from the seafloor, which contribute to climate change. Since China entered the 13th Five-Year Plan, on the one hand China’s marine fisheries have begun to pay attention to green development, and on the other hand trawling has mainly been used to catch benthic organisms such as shrimp and crab, and seawater shrimp and crab farming have gradually replaced marine fishing as the main source of seawater products, so there was a downward trend after 2015. Gillnetting accounts for approximately 26.37% of the total CO_2_ produced by the marine fishing industry, with an overall increasing trend, with 61.28% in 2016 compared to 2005, followed by a decreasing trend year by year.

**Figure 5:**
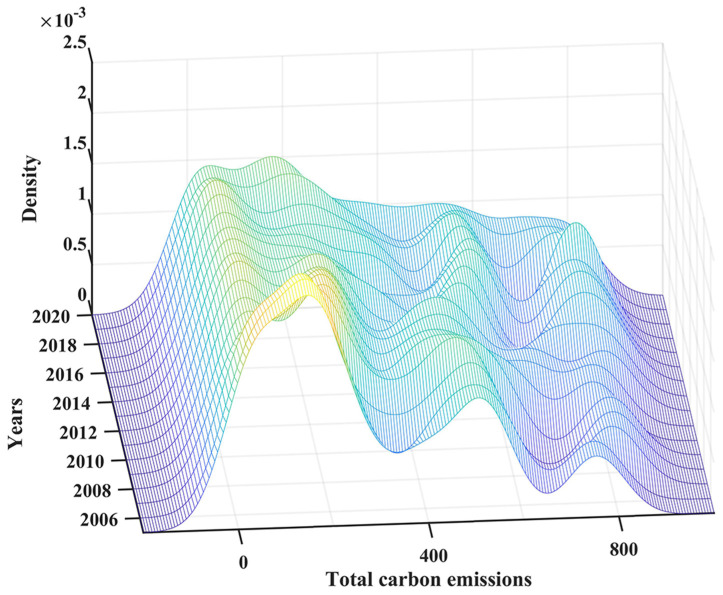
Kernel density curve of marine fisheries’ CO_2_.

Figure 5 presents the kernel density of total carbon emissions from marine fisheries for 2005–2020. From the position of the curve, the center of the kernel density curve shifts first to the right and then to the left, indicating a gradual increase in marine fisheries’ CO_2_ emissions over the study period, followed by a decrease. In respect to the extension of the curve, the curves exhibit an obvious skewed distribution and multimodal distribution, which is indicative of the uneven development of carbon emissions from marine fisheries and an apparent polarization feature. Additionally, the kernel density corresponding to the first wave peak is higher than the subsequent waves, indicating that the proportion of provinces with fewer carbon emissions was greater than the proportion of provinces with higher emission levels during this period. The Kernel density curve changed from being “Sharp and narrow” to being “Flat and wide,” suggesting that the general gap between coastal provinces tended to narrow from 2005 to 2013. After that, it gradually widened.

#### 4.1.2. Spatial Variability Characteristics

**Figure 6:**
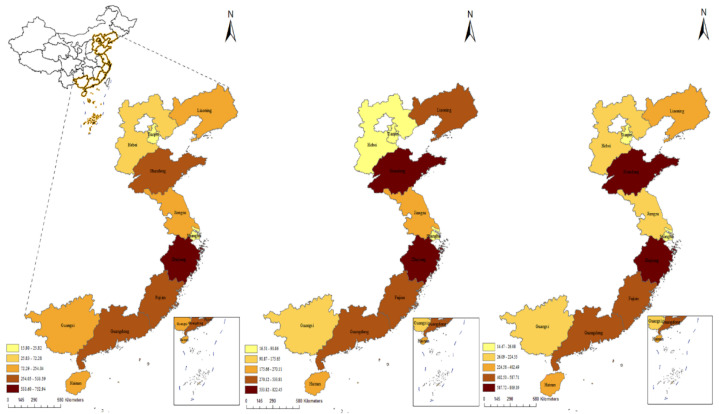
Spatial and temporal distribution of carbon emissions from marine fisheries in 11 coastal provinces of China; the graphs represent 2005 (**left**), 2012 (**middle**), and 2020 (**right**), respectively.

Marine fisheries’ CO_2_ emissions were classified into five classes based on the natural breakpoint method to explore the spatial pattern characteristics of China (Figure 6). It can be seen that the CO_2_ emissions are unevenly distributed throughout the provinces in terms of regional distribution, there is some spatial variation, and the economically developed provinces present worse patterns than less developed ones. In 2005, China’s marine fisheries remained at the level of the unilateral pursuit of economic expansion, which led to a high number of energy-intensive fishing boats releasing significant quantities of greenhouse gases. Carbon emissions decreased in Hebei and Guangxi in 2012, but continued to grow in other regions; however, until 2020 Zhejiang Province had the greatest CO_2_ emissions from 2005 to 2020 due to the consistently highest inshore fishing intensity and productivity. The main operation methods of Zhejiang’s marine fishing industry are carbon-intensive trawling and gillnetting, and trawlers remained popular, all of which contribute to the long-term high carbon status of marine fisheries.

### 4.2. Test Results

#### 4.2.1. Autocorrelation Test Results

**Figure 7:**
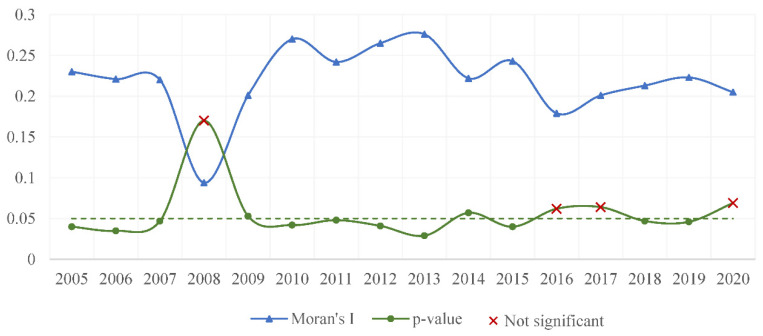
Autocorrelation test results.

Figure 7 depicts the global autocorrelation coefficients, and it can be seen that the *p*-values for the vast majority of years are positive and pass the 0.05 significance level test, indicating a positive spatial correlation of carbon emissions among coastal provinces.

#### 4.2.2. The LM, LR, and WALD Tests

After passing the autocorrelation test, the LM test was used to determine whether an OLS model or a spatial econometric model should be used. According to the results of the LM test, the *p*-values are all significant at the 1% level, so the OLS model should be rejected and the spatial econometric model should be chosen; we assumed that the SDM in the spatial econometric model was the optimal model for the validation and analysis of spatial effects, and then we utilized the LR test to evaluate if the SDM could degenerate into the SEM or SLM. The results of the LR test are shown in Table 3. We can reject the original hypothesis, since the test findings are significant at the 1% level, which indicates that the SDM will not degenerate into the SEM or SLM. Therefore, the SDM was finally chosen to study the spatial effects of carbon emissions from marine fisheries in China.

### 4.3. Estimation Results

#### 4.3.1. Spatial Measurement Results

This study used the spatial fixed effect model to test the impact of socioeconomic variables on carbon emissions from marine fisheries. Considering that the SDM can be expanded into a province fixed effect (Column 1 in Table 4), time fixed effect (Column 2 in Table 4), and double fixed effect (Column 3 in Table 4), the regression was conducted separately, and the double fixed effect with the largest R-squared was selected as the final model.

The regression coefficients of *lnGMFP* and *lnIMEX* were all significantly positive, which indicates that the growth of the marine fishery economy and the expansion of seafood trade would increase CO_2_ emissions. The demand for economic growth and export earnings motivates fishery enterprises to scale up production; furthermore, the expansion of the fishery industry could lead to changes in the life activities and production of fish and other marine animals, resulting in a continuous increase in CO_2_. In contrast to the majority of studies, the coefficient for the optimization of industrial structures is also significantly positive at the 10% level, revealing that the increase in the tertiary sector of the marine fishery industry does not reduce CO_2_ emissions and that the general marine fishery industry in China remains resource-intensive.

Technological innovation and an increase in the net income of fishermen can significantly reduce CO_2_ emissions. Increased internal spending on R&D has fostered scientific fishing and farming developments, as well as the emergence and application of low-carbon and environmentally friendly technologies. As opposed to most econometric estimates, the coefficient of the fishermen’s net income variable is negative, which could be explained by the decreasing proportion of household income in the net income of Chinese fishermen and the gradual increase in net property income, which reduces the incidence of carbon emission production activities. It is noteworthy that *lnPOL* failed the statistical significance test, suggesting that China’s “ fishing vessels reduction and power reduction” policy has had no significant impact on the environment.

#### 4.3.2. Robustness Tests

In considering the fact that the regression results are sensitive to the selection of spatial weights, this paper verifies the robustness of the conclusions using the two other spatial weight matrices. One is the economic spatial weight matrix, where the non-diagonal elements of the matrix are the inverse of the absolute value of the difference between the real GDP per capita of the two regions, and the diagonal elements are zero, for which the formula is:

(13) $$W_{ij}^{GDP}=\{\begin{array}{c} \frac{1}{\left|\begin{array}{c} Y_{i} \end{array}-Y_{j}\right|}\;\mathrm{if}\;i\neq j\\ 0\;\mathrm{if}\;i=j \end{array}$$

The other is the spatial geographic distance weight matrix, which is constructed based on the geographic distance between regions with the following equation:

(14) $$W_{ij}^{DD}=\{\begin{array}{c} \frac{1}{d_{ij}^{2}}\;\mathrm{if}\;i\neq j\\ \;0\;\mathrm{if}\;i=j \end{array}$$

It should be noted that the spatial weight matrices included in the empirical analysis are all row-standardized to eliminate the effect of measurement units. When the aforementioned two spatial weight matrices are replaced, the findings suggest that spatial correlation still exists between provinces (as shown in columns 4 and 5 of Table 4), and both of them pass the LM, LR, and WALD tests at a 1% level, although the R-squared is relatively small.

Another commonly used robustness test is Winsorizing: in a Winsorized estimator, the extreme values are instead replaced by certain percentiles [54,55]; we subject all the variables to a top and bottom 1% tail shrinkage. As can be observed in column 6 of Table 4, the only difference between the robustness test and the baseline regression findings is the magnitude of the coefficients; the directions of effect and significance are identical.

#### 4.3.3. Spatial Spillover Effect Analysis

The spatial econometric model can show the spatial correlation characteristics among regions, but the regression results of the SDM do not fully reflect the effect of the explanatory variables on the explained variables, and changes in the independent variables not only affect the dependent variables in the region (spatial feedback effect) but also may affect the dependent variables in other regions (spatial spillover effect). Lesage and Pace [46] used partial differencing to specifically decompose the effects of the explanatory variables on the explained variables into direct effects, indirect effects, and total effects. The decomposition results of the respective variables in this paper are shown in Table 5, and the “*Indirect*” column indicates the spillover effect.

The spatially lagged regression coefficient of *lnGMFP* is notably negative at the 5% level, showing that the growth of the marine fishery economy exacerbates local carbon emissions but decreases carbon emissions in neighboring provinces through spatial spillover effects. This is primarily due to the agglomeration and polarization effects of China’s fishery economy. The more economically developed the provinces are, the higher level of market demand and the greater factor returns; consequently, the production factors become more concentrated in economically developed areas, thereby reducing fishing activities in less economically developed regions.

The coefficient of industrial structure optimization is positive at the 1% level, suggesting that industrial restructuring would boost carbon emissions in neighboring provinces. China’s marine aquatic product processing industry has not yet transitioned to a technology-intensive stage, and material and human capital inputs and resource losses are still increasing continuously. Thus, provinces have not been able to effectively collaborate on the division of labor and industrial synergy, which at this point would only result in imitation behavior, thus leading to an increase in CO_2_ in neighboring provinces.

The estimation result of *lnPIC* expresses that increased income for fishermen decreases local carbon emissions while increasing emissions in neighboring regions. High-income areas have a diminishing marginal propensity to pollute, whereas governments in low-income areas are more likely to develop the economy at the expense of the environment; income disparity affects people’s expectations, resulting in labor outflow from low-income areas, thereby impeding the improvement in labor quality in low-income areas. Meanwhile, an increase in low-quality labor would inhibit technological progress and weaken the incentive for industrial structure development in underdeveloped coastal provinces, thus further perpetuating high-carbon fishing production activities.

## 5. Conclusions

The purpose of this study was to examine the carbon emissions of China’s marine fishery industry, a sector that accounts for a relatively small share of the entire industry yet has a far-reaching influence on the marine environment. Kernel density estimation, the spatial autoregressive test, SDM estimation, SDM results decomposition, the replacement of the spatial weight matrix, and removal of extreme values are the panel econometric techniques that were used in this study. Based on the results of the empirical analysis, the following conclusions were obtained: (1) the marine fishing industry is the biggest source of CO_2_ emissions, contributing approximately 72% of total emissions, and trawling is the most CO_2_-emitting marine fishing practice; (2) according to kernel density estimation, the total CO_2_ emissions from marine fisheries in China’s coastal regions are uneven and the gap is gradually widening; (3) from empirical evidence, the SDM concludes that the economic scale is enlarging, which includes an increase in gross marine fishery production, the expansion of the tertiary sector, and the import and export of seafood, all of which contribute to a rise in CO_2_ emissions; China’s long-standing strategy of “ fishing vessels reduction and power reduction “ has had no discernible impact, according to our research; and (4) additionally, there is a significant spillover effect of carbon emissions from marine fisheries among coastal provinces in China; marine fishery economic development aggravated local CO_2_ emissions while reducing them in neighboring provinces; income for fishermen lessens local carbon CO_2_ emissions while increasing them in neighboring areas; industrial restructuring also increases carbon spillover.

## 6. Policy Implications and Future Research

The following are the sustainable development policy implications of this research. Firstly, the contribution of mariculture and marine aquatic product processing is gradually increasing; therefore, the energy-intensive production activities in these two sectors should be restrained, and governments are urged to take action. Additionally, when adopting marine strategies, the Chinese government should take into consideration the effectiveness of the policy environment. Furthermore, they should encourage and promote breakthrough environmental technology, since this plays an essential role in this field. The industrial structure is an important factor affecting marine fishery emissions. We found that the enhancement of the tertiary industry simultaneously aggravates local CO_2_ emissions and the environmental degradation of neighboring regions by spatial spillover effects. To achieve an ecologically friendly and low-carbon service industry growth model, it is vital the internal structure of the tertiary sector is optimized. Finally, the fishery administration should coordinate interprovincial interaction in emission reduction, build a diversified production factor exchange mechanism, and speed up the promotion of carbon reduction through the division of labor among neighboring provinces to prevent pollution migration.

The spatial econometric model measurement findings may only offer a partial view. A more disaggregated sectoral examination, more comprehensive socioeconomic factors, and how responsibilities are distributed among the coastal provinces need to be followed up with further examination. Furthermore, fisheries’ carbon sinks in the assessment framework should be considered in future work.

## Figures and Tables

**Table 1:** The meaning of the symbols in the formula.

Symbol	Meaning	Symbol	Meaning
$TC_{fishery}$	Total carbon emissions of marine fisheries	*i*	Coastal provinces of China
$C_{mf}$	Carbon emissions from marine fishing	*t*	Study period from 2005 to 2020
$C_{mc}$	Carbon emissions from mariculture	r	Net calorific value of diesel fuel
$C_{mp}$	Carbon emissions from marine aquatic products processing	f	Conversion factor of diesel fuel to standard coal
$E_{ijt}^{diesel\_mf\;}$	Total energy (diesel) consumed by marine fishing	$\theta_{i}$	Coefficient of electric power conversion to CO_2_ of each province
$E_{it}^{power\_mf\;}$	Total power of marine fishing vessels	o	Oxidation rate of diesel combustion
$E_{it}^{diesel\_mc\;}$	Total energy (diesel) consumed by mariculture fishing vessels	c	Carbon content per unit calorific value of diesel fuel
$E_{it}^{electric\_process}$	Electricity consumption during seafood processing	$\delta^{mc}$	Energy consumption coefficient of marine aquaculture fishing vessel operation
$E_{it}^{electric\_cold\_store}$	Electricity consumption during seafood freezing	$\delta_{j}^{mf}$	Energy consumption coefficient of different operation modes of marine fishing vessels
$E_{it}^{electric\_mc\;}$	Power consumption of oxygen supply and electric pump during mariculture ponds and industrial farming.	*p*	Operation mode of marine fishing vessels: trawling, purse seining, gill netting, spread netting, fishing gear, and others

**Table 2:** Explanation of variables.

	Variable Name	Calculation Process
Dependent variable	Carbon emissions per capita of marine fisheries *(lnPCO_2_*)	Total carbon emissions from marine fisheries divided by marine fishery population
Independent variable	Marine fishery economic development *(lnGMFP)*	Gross marine fishery product
	Marine fishery industry structure optimization (*lnINDUS)*	Marine fishery tertiary industry divided by secondary industry
	Marine fishery technology innovation *(lnTECH)*	Internal expenditure of R&D funds for marine fishery science and technology promotion
	Increase in fishermen’s income (*lnPCI*)	Fishermen’s net income per capita
	Deepening of seafood trade (*lnIMEX*)	Total import and export of marine fishery
	Environmental regulation *(lnPOL)*	Proportion of “ reduced vessels and reduced power” fishing vessels to total fishing vessels

**Table 3:** The LM, LR, and WALD test results.

	Test Method	Statistics	*p*-Value
LM test	Spatial error		
	Moran’s I	2.09	0.037
	Robust LM	16.217	0.000
	Spatial lag		
	Robust LM	22.666	0.000
LR test	SAR nested within SDM		
	LR ${\mathrm{chi}}^{2}$(6)	19.92	0.0029
	SEM nested within SDM		
	LR ${\mathrm{chi}}^{2}$(6)	15.99	0.0138
WALD test	SAR ${\mathrm{chi}}^{2}$(6)	21.13	0.0017
	SEM ${\mathrm{chi}}^{2}$(6)	16.46	0.0115

**Table 4:** Estimated results and robustness tests.

*lnPCO_2_*	(1)	(2)	(3)	Robustness Tests		
				$(4)\;W_{ij}^{DD}$	$(5)\;W_{ij}^{GDP}$	(6) Winsorize
*lnGMFP*	0.115 **	−0.123 ***	0.121 **	0.131 **	0.0358	0.116 **
	(0.0528)	(0.0206)	(0.0497)	(0.0548)	(0.0267)	(0.0505)
*lnINDUS*	0.138 **	0.121	0.128 *	0.124 *	0.0598	0.123 *
	(0.0657)	(0.104)	(0.0656)	(0.0683)	(0.0787)	(0.0670)
*lnPIC*	−0.351 ***	0.289 *	−0.430 ***	−0.463 ***	−0.469 ***	−0.423 ***
	(0.109)	(0.155)	(0.114)	(0.117)	(0.116)	(0.120)
*lnTECH*	−0.0335 ***	0.0514 ***	−0.0261 **	−0.0246 **	−0.0418 ***	−0.0250 **
	(0.0116)	(0.0140)	(0.0124)	(0.0120)	(0.0130)	(0.0125)
*lnPOL*	−0.103	−0.998 ***	−0.0240	−0.641 *	−0.0244	−0.0298
	(0.328)	(0.361)	(0.330)	(0.332)	(0.329)	(0.336)
*lnIMEX*	0.0653 *	0.0636 **	0.108 ***	0.0612	0.0731 **	0.103 ***
	(0.0355)	(0.0319)	(0.0355)	(0.0396)	(0.0347)	(0.0360)
*W × lnGMFP*	−0.0185	−0.394 ***	−0.0131	0.206	−0.172 ***	−0.0261
	(0.121)	(0.103)	(0.138)	(0.131)	(0.0649)	(0.143)
*W × lnINDUS*	0.323 ***	0.195	0.256 *	0.193	0.133	0.266 *
	(0.0979)	(0.199)	(0.147)	(0.166)	(0.194)	(0.149)
*W × lnENERGY*	0.533 ***	2.864 ***	−0.0919	0.137	−0.272	−0.0227
	(0.183)	(0.314)	(0.324)	(0.286)	(0.392)	(0.344)
*W × lnTECH*	−0.00504	−0.0246	−0.00506	0.0268	−0.0193	−0.00440
	(0.0206)	(0.0349)	(0.0343)	(0.0319)	(0.0368)	(0.0347)
*W × lnPOL*	0.206	−2.170 ***	0.126	−2.087 ***	0.834	0.123
	(0.506)	(0.746)	(0.605)	(0.711)	(0.697)	(0.616)
*W × lnimex*	0.129	−0.301 ***	0.366 ***	0.168 *	0.361 ***	0.346 ***
	(0.0925)	(0.111)	(0.100)	(0.0859)	(0.119)	(0.101)
ρ	−0.353 ***	−0.00680	−0.536 ***	−0.516 ***	−0.420 ***	−0.526 ***
	(0.109)	(0.106)	(0.107)	(0.104)	(0.133)	(0.107)
Province effect	Yes	No	Yes	Yes	Yes	Yes
Year effect	No	Yes	Yes	Yes	Yes	Yes
R-squared	0.209	0.302	0.539	0.2218	0.035	0.536

Note: Standard errors in parentheses; *** *p* < 0.01, ** *p* < 0.05, * *p* < 0.1.

**Table 5:** Decomposition results.

*lnPCO_2_*	Direct Effect	Indirect Effect (Spillover Effect)	Total Effect
*lnGMFP*	0.000396 **	−0.000905 **	−0.000509
	(0.000201)	(0.000387)	(0.000391)
*lnINDUS*	0.0628	0.229 ***	0.292 ***
	(0.0692)	(0.0850)	(0.0720)
*lnPIC*	−0.439 ***	0.832 ***	0.393 ***
	(0.117)	(0.180)	(0.137)
*lnTECH*	−0.0316 ***	−0.00217	−0.0338 **
	(0.0122)	(0.0185)	(0.0165)
*lnPOL*	−0.0813	0.0841	0.00277
	(0.367)	(0.472)	(0.485)
*lnIMEX*	0.0792 **	0.0204	0.0996
	(0.0396)	(0.0865)	(0.0890)

Note: Standard errors in parentheses; *** *p* < 0.01, ** *p* < 0.05, * *p* < 0.1.

## Data Availability

The data presented in this study are available on request from the corresponding author. The data are not publicly available due to privacy.
